# Deep learning for synovial volume segmentation of the first carpometacarpal joint in osteoarthritis patients

**DOI:** 10.1016/j.ostima.2024.100176

**Published:** 2024-03-04

**Authors:** Carla du Toit, Megan Hutter, Igor Gyacskov, David Tessier, Robert Dima, Aaron Fenster, Emily Lalone

**Affiliations:** a Robarts Research Institute, Western University, London, Ontario, Canada; b Department of Medical Biophysics, Western University, London, Ontario, Canada; c Department of Mechanical and Materials Engineering, Western University, London, Ontario, Canada; d Department of Health Sciences, Western University, London, Ontario N6A 5B7, Canada

**Keywords:** Synovial membrane volume segmentation, 3D ultrasound imaging, First carpometacarpal osteoarthritis, Deep learning, Convolutional neural networks

## Abstract

**Objective:**

The objective of this study was to develop a deep-learning-based approach to automatically segment 3D ultrasound images of the synovial tissue in osteoarthritis of the first carpometacarpal (CMC1 OA).

**Design:**

Deep learning predictions on 2D ultrasound slices sampled in the transverse plane were used to view the synovial tissue of the first carpometacarpal in patients with OA, followed by reconstruction into 3D surfaces. A modified 2D U-Net was trained using a dataset of 832 2D US images resliced from 89 3D US images. Segmentation accuracy was evaluated using a testing dataset of 208 2D US images resliced from 15 3D US images. Absolute and signed performance metrics were computed, and segmentation performance was compared between the manual segmentations of raters 1 and 2.

**Results:**

Results of the U-Net-based run were mean 3D DSC 86.9 ± 4.8%, recall 93.7 ± 3.6%, precision 81.1 ± 6.9%, volume percent difference 16.9 ± 10.2%, mean surface distance 0.18 ± 0.04 mm, and Hausdorff distance 1.8 ± 0.8 mm. The algorithm demonstrated an overall increase in performance after 3D segmentation reconstruction compared to 2D predictions, but the difference was not statistically significant.

**Conclusion:**

This study investigated the use of a modified U-Net algorithm to automatically segment the synovial tissue volume (STV) of CMC1 OA patients and demonstrated that the addition of this deep learning method increases the efficiency of STV estimations in clinical trial settings.

## Introduction

Osteoarthritis (OA) of the first carpometacarpal (CMC1) is a progressive, multifactorial disease with a symptomatic prevalence of approximately 15% of those 30 years or older and a current radiographic prevalence of 90% in individuals over the age of 80 [1]. OA affects all tissues of the CMC1 joint and is associated with pathologies such as cartilage degradation, changes in the muscular and ligamentous integrity, abnormal subchondral bone remodelling, angiogenesis, and synovitis [[2], [3], [4]].

Synovitis is associated with increased pain and disability in patients with OA and is an important factor influencing disease progression [5]. Chronically elevated levels of inflammatory mediators associated with synovitis lead to cartilage degeneration and alterations of the underlying subchondral bone [[5], [6], [7]]. Synovial effusion and membrane hypertrophy are frequently observed by magnetic resonance imaging (MRI) and 2D ultrasound imaging (2D US) [8].

Measures of synovitis and synovial membrane hypertrophy are integral for monitoring CMC1 OA disease progression and treatment planning. The Outcome Measures in Rheumatology (OMERACT) is the most common semi-quantitative grading scale for characterizing synovitis using 2D US. [9] Currently, radiographic characterization of CMC1 OA is based on the Eaton-Littler grading scale, which uses measures of joint space narrowing, cyst formation, sclerosis, the presence of osteophytes, and joint subluxation [10,11]. Although radiographic grading is most commonly used to characterize and monitor CMC1 OA disease progression, it lacks sensitivity to changes in synovitis and surrounding structures due to insufficient soft tissue contrast . The discrepancy between radiographic evidence of OA and patient-reported symptoms is well-reported in the OA literature [12], [13], [14].

3D US is an attractive alternative imaging technique that allows quantitative and qualitative characterization of synovitis in CMC1 OA patients. It has also been investigated widely for applications in cardiology, urology, neonatology, gynecology, and more recently in rheumatology [15], [16], [17], [18]. The addition of 3D US imaging to clinical applications could provide users with easily accessible, time-efficient methods for assessing CMC1 OA disease status and progression. Our lab has developed a 3D US device specifically for imaging of the hand and wrist.

Currently, segmentation of joint synovitis from 3D US images and quantifying its volume is essential for broadening our understanding of the disease and providing a comprehensive characterization of the patient-specific disease profile influencing clinical tasks including treatment planning and monitoring of response. A highly trained rater typically performs tissue segmentation manually. This process is time-consuming, as segmentations have to be completed on each 2D image slice. Segmentations require approximately 20 min per 3D US image. Efficiency and repeatability vary as inter- and intra-rater reliability is influenced by the user's level of expertise [18,19].

Convolutional neural networks (CNNs) have shown promising results for segmenting cartilage, bone, and synovial tissue in knee osteoarthritis patients using MRI, 2D US, and 3D US [[20], [21], [22], [23]]. However, there is a distinct lack of exploration of deep learning networks for measuring and identifying characteristics of OA in the small joints of the hands. Schwartz et al. reported that CNN methods can identify and classify knee OA from radiographs as accurately as a fellowship-trained arthroplasty surgeon[24]. A study by Wu et al. showed that deep-learning models could be used to evaluate the automatic classification of rheumatoid arthritis in metacarpophalangeal joints using ultrasound. They used a DenseNet-based deep learning model and assessed the area under the curve (AUC), accuracy, sensitivity, and specificity to identify rheumatoid arthritis using OMERACT-EULAR parameters on 2D US. Their highest reported accuracy rate was 82.1% [25].

In a previous study conducted by our lab, we investigated the application of U-Net and U-Net++ networks to segmentations of femoral articular cartilage in knee osteoarthritis patients [21]. These networks were trained using 200 2D US slices obtained from 20 3D US volumes. The study reported an average Dice similarity coefficient (DSC) score of 82.3 ± 6.1% for the cohort of knee OA patients.

Based on previous studies that established the concurrent validity between the imaging standard of MRI and the 3D US device used in this study, we maintain the assumption that the 3D US-based measurements of synovial volume are comparable to the diagnostic reference standard [26,27]. We hypothesize that adding deep learning segmentation ability to the current 3D US imaging system will provide operators with fast and accurate 3D segmentations of CMC1 OA synovial tissue volumes (STV) in 3D US images of the CMC1 joint.

The aim of this work is to demonstrate that a 2D deep learning model plus a 3D reconstruction approach can effectively produce the STV required for disease monitoring. This was determined by comparing the performance of our U-Net++ algorithm to a standard U-Net algorithm and manual segmentation created by trained raters. Performance metrics such as the Dice Coefficient, recall accuracy, volume percent difference and differences in boundary distances in the images will provide insight into the performance capabilities of the algorithm.

## Materials and methods

### Clinical data set

**Fig. 1:**
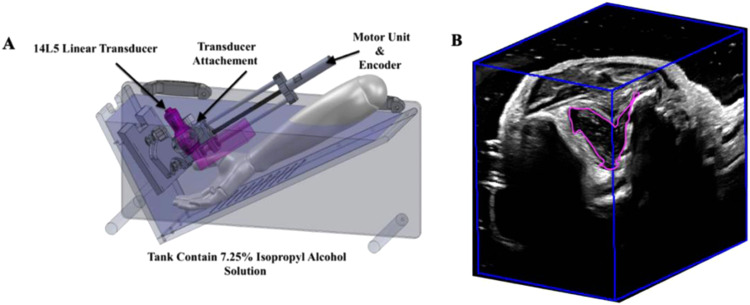
A schematic of the semi-submerged 3D US scanning device (A). An example of the resulting 3D US image with the synovial tissue membrane segmented in purple (B).

Twenty CMC1 OA patients over the age of 18 were recruited from the Roth McFarlane Hand and Upper Limb Clinic for 3D US imaging (Table 1). The imaging protocol was approved by the Research Ethics Board at Western University, Canada, and all participants provided written consent prior to imaging. Bilateral 3D US images of CMC1 joints were acquired four times for these OA patients (at baseline, 3 months, 6 months, and 1 year) using our 3D US device. This device and its mover unit are compatible with any commercially available ultrasound machine and transducer through custom-made mover holders and image acquisition software. For this study, our device was connected to a Canon Aplio i700 US machine (Canon Medical Systems, Tustin, California, US) and used a L14–5 linear transducer with a 10 MHz operating frequency (3.8 MHz – 10.0 MHz) and 58 mm footprint to image the CMC1 using a linear mechanical scanning approach (Fig. 1) [28]. To obtain these 3D US images the transducer was translated up to 30 cm, depending on the size of the patient's hand. This transducer was attached to a semi-submerged 3D US imaging device via a custom 3D printed mount. As the transducer was translated, the 3D US imaging system acquired 336 2D US images, which were spaced at regular spatial intervals, and the resulting images were reconstructed into a 3D US image (0.114×0.114×0.333 mm [3] voxel size) as the images were acquired, resulting in 3D US images available immediately after completion of the scan As shown in Fig. 1, patients were scanned through the thenar eminence on the volar side of the hand allowing for increased visualization of the CMC1 joint. Manual segmentations of synovitis and synovial hypertrophy were created by two raters to generate the dataset used for training and testing the deep learning model. Rater 1 had 3 years of experience in musculoskeletal ultrasound research while rater 2 had 1 year experience.

**Table 1:** Demographic information for the first carpometacarpal osteoarthritis cohort.

	CMC1 OA Patients
N	20
Sex Ratio (Female: Male)	13:7
Age [years] (mean ± SD)	66.1 ± 6.5

### Deep learning training dataset

The twenty patients received bilateral 3D US scans in four imaging sessions over a twelve-month period. The total data set consisted of 104 3D US imaging studies after the exclusion of studies that contained no synovitis. Each of the consecutive images differed in scan orientation, anatomical view (volar, dorsal, lateral), and hand-sidedness in addition to changes in the patient's joint anatomy as a result of the progression of pathology. A region of interest was manually established around the CMC1 joint in each of these images, which consisted of a boundary approximately 15 pixels wide in the X, Y, Z directions. The regions-of-interest of the 3D US images were each sliced into 8–15 2D US slices to give a dataset of 1040 2D US. The resulting dataset was further split into approximately 80% for training (89 3D US images generating 832 2D images) and 20% for testing (15 3D US images generating 208 2D images). These 2D US slices were placed back into the 3D volume in known locations to create the 3D US surfaces.

### Deep learning model and 3D reconstruction

The widely used U-Net architecture and its variants have demonstrated high performance in various medical image segmentation tasks [29,30]. Our lab has previously used the U-Net approach and applied it to trochlear cartilage thickness segmentation of the knee using 3D US images of patients with knee osteoarthritis [21]. The U-Net model was implemented using Keras with a TensorFlow backend and made use of the Adam optimizer, a 1 × 10−4 learning rate, 100 epochs, and 600 steps per epoch [31]. Additionally, our U-Net++ model used a ResNet-18 network with batch normalization of 10. To artificially expand the training dataset, data augmentation was implemented, which consisted of random combinations of vertical and horizontal flips, rotations up to 20º, translations up to 20%, and zooms up to 20%. Neural network training and inferencing were completed on a personal computer with an i7–9700 K CPU running at 3.60 GHz (Intel Corporation, Santa Clara, CA, USA), 64 GB of RAM, and a 24 GB NVIDIA TITAN RTX (NVIDIA Corporation, Santa Clara, CA, USA) GPU. The resulting 2D predictions were placed back in their original position within the 3D volume and the points on the segmented boundaries of adjacent slices were connected. Once this process was complete the ends of the volume were closed resulting in the complete 3D surface.

### Evaluation metrics and comparisons

To evaluate our method's performance, 2D segmentations and the reconstructed 3D surfaces were used to compute standard pixel map metrics including, DSC, precision, and recall. These metrics were calculated for each 2D US image using these confusion matrix values: false positive — surface area /volume labelled as synovitis in the algorithm but not in the gold standard; true positive — overlapping synovitis surface area/volume between the gold standard manual and the algorithm segmentations; false negative — surface area/volume identified as synovitis in the gold standard that was missed by the algorithm; and the true negative — surface area/volume identified as not synovitis in both the gold standard and algorithm. Values from the confusion matrix for each 2D US slice were averaged to find the recall, precision, and accuracy measurement for the 3D US reconstruction. Additionally, absolute and signed variants of area/volume percent difference and boundary distance metrics including mean surface distance, and Hausdorff distance were computed. All evaluation metrics aside from the 3D boundary distance metrics were computed using custom software in MATLAB R2022a (MathWorks, Natick, MA, United States). 3D mean surface distance and Hausdorff distance were computed using CloudCompare (v2.10.2, Girardeau-Montaut). 2D slice segmentation time, reconstruction time, and overall 3D segmentation time for our algorithm were recorded.

### Statistical analysis

All statistical analyses were completed using GraphPad Prism (GraphPad Software, Inc., San Diego, CA, USA). The distribution normality of the data was analyzed using a Shapiro-Wilk test and the significance level was established so that the probability of making a type 1 error was less than 5% (*p* < 0.05). The comparisons of segmentation accuracy between the manual and U-Net/U-Net++ were completed for both 2D and 3D US metrics using a two-tailed paired *t*-test. When the established normality assumptions were violated, a Wilcoxon matched-pairs signed-rank test was used instead. CMC1 STV segmentation performance using U-Net compared to rater 1 and 2 manual segmentations was compared using two-tailed paired *t*-tests or Wilcoxon matched-pairs signed-rank tests. Bland-Altman plots were used to assess the distribution and the bias of the absolute STV differences between manual STV segmentations and the two different algorithm-based (U-Net and U-Net++) reconstructed STV. Additionally, these plots were also used to assess the distribution and fixed bias of the absolute STV differences between the two raters as seen in Fig. 4.

## Results

**Fig. 2:**
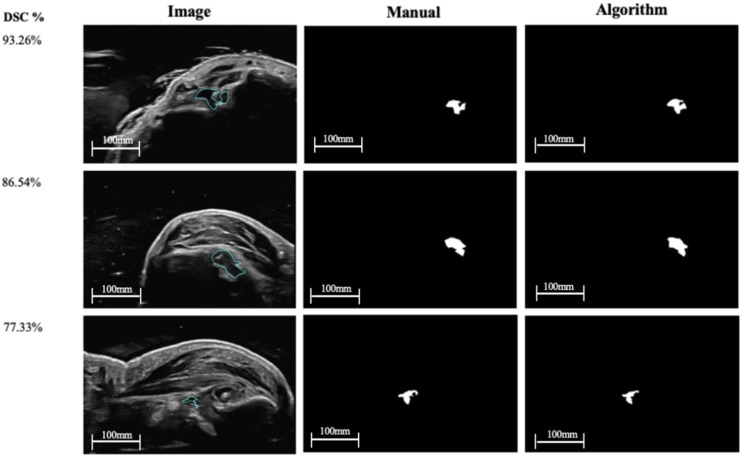
Examples of first carpometacarpal synovial tissue segmentation results using the U-Net model. The original 2D US images are in the left column, the manual segmentation masks are in the middle and the algorithm-generated segmentation masks are on the right. Each row represents varying performance levels based on the DSC metric with values of 93.3% for the top, 86.5% for the middle, and 77.3% for the bottom row.

**Fig. 3:**
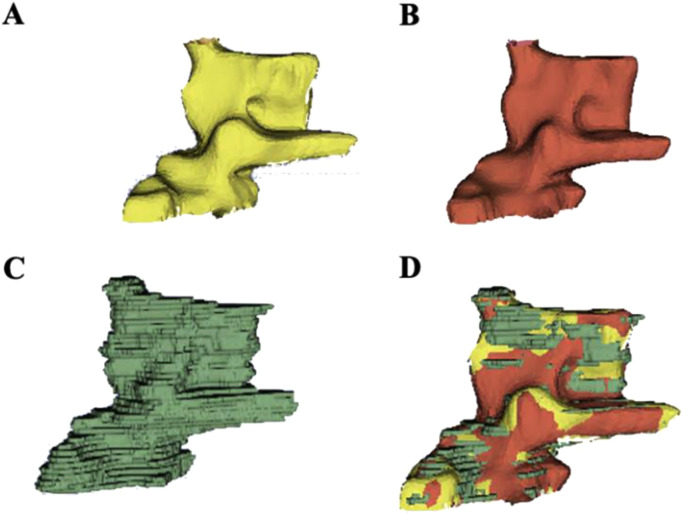
(A) Resultant 3D reconstructed surfaces using the 2D segmentations generated by (A) U-Net, (B) U-Net++, and (C) rater 1 for a CMC1 OA patient. (D) Registered manual and algorithmic segmentations overlayed in 3D Slicer.

Fig. 2 shows three 2D US segmentation results (masks) at three different DSC performance levels ranging from 77% to 93%. The manual and algorithm segmentation results are shown in the same spatial location as the 2D US images, allowing visual evaluation. Fig. 3 shows a comparison between the 3D reconstructed surfaces from the 2D segmentations generated manually, with U-Net, and with U-Net++ for a segmentation DSC of 87.32%.

**Fig. 4:**
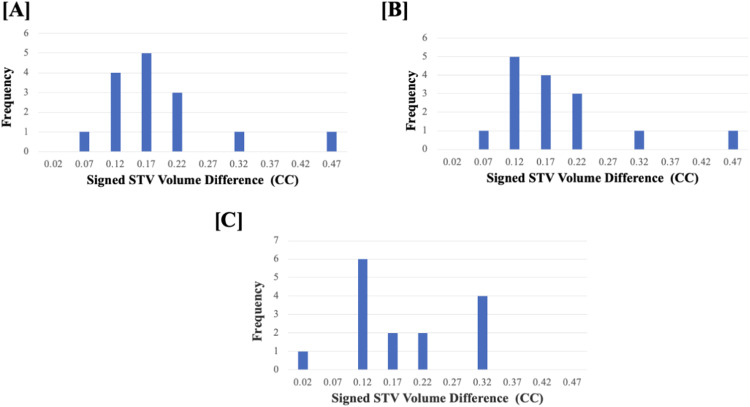
(A) Distribution and (B) fixed bias of the absolute STV difference of the manual STV segmentations compared to the U-Net reconstructed STV. (C) Distribution and (D) fixed bias of the absolute STV difference of the manual STV segmentations compared to the U-Net++ reconstructed STV. (E) Distribution and (F) fixed bias of the absolute STV difference of the manual STV segmentations of rater 1 compared to rater 2.

Tables 2 and 3 show the absolute and signed performance metric results for the segmentations on the 2D slices and the resulting 3D reconstructions using U-Net and U-Net++. Fig. 4 depicts the distribution and fixed bias of the absolute STV difference of the manual STV segmentations compared to the U-Net and U-Net++ reconstructed STV. Additionally, Fig. 4 depicts the distribution and fixed bias of the absolute STV difference of the manual STV segmentations completed by rater 1 and rater 2. For the segmentations on the 2D slices, all performance metrics indicated that there was a statistically significant difference between U-Net and U-Net++. Performance metrics assessed for the 3D synovial volume reconstructions indicated that there were no statistically significant differences between U-Net and U-Net++, except for Hausdorff distance. Overall, the results indicated that there was an increase in performance after 3D reconstruction. Decreased performance was observed in precision, accuracy, and Dice coefficient and increased performance was seen in recall, mean surface distance and area/volume percent difference when comparing the 2D slice segmentations and the 3D reconstructions. However, a Wilcoxon matched-pairs signed-rank test indicated that only changes in precision, accuracy and Hausdorff distance were statistically significant. The U-Net++ outperformed U-Net on all the tested performance metrics. When comparing the resultant volume measurements to those done manually, both the U-Net and U-Net++ indicated lower percent differences for the segmentations on the 2D slices than for the 3D reconstructions. However, neither of these differences was statistically significant when tested using the Wilcoxon matched-pairs signed-rank test.

**Table 2:** Mean and standard deviation performance metric results comparing synovial tissue segmentations using 2D U-Net to 2D U-Net++. Also, the performance metric comparing the 3D reconstructions of the synovial volume based on the 2D U-Net and 2D U-Net++. P-values correspond to a comparison between U-Net and U-Net++ segmentation performance for the 2D and 3D segmentations respectively.

Segmentation	Method	Precision (%)	Recall (%)	Accuracy (%)	DSC (%)	MSD (mm)	HD (mm)	A/VPD (%)
2D Images	U-Net	82.3 ± 10.4	87.6 ± 8.9	92.9 ± 3.3	84.3 ± 7.2	0.35 ± 0.2	3.2 ± 1.7	52.2 ± 45.2
	U-Net++	85.6 ± 8.4	93.1 ± 5.4	99.8 ± 0.1	88.9 ± 5.4	0.22 ± 0.11	1.1 ± 0.74	36.2 ± 40.05
	P-value	0.04	0.0001	0.0001	0.0001	0.0001	0.0008	0.006
3D Reconstructed Images	U-Net	81.1 ± 6.9	93.7 ± 3.6	96.2 ± 1.5	86.9 ± 4.8	0.18 ± 0.04	1.8 ± 0.8	16.9 ± 10.2
	U-Net++	81.1 ± 6.8	93.7 ± 3.6	96.16 ± 1.5	87.8 ± 0.1	0.18 ± 0.03	0.5 ± 0.1	16.8 ± 10.3
	P-value	0.16	0.19	0.33	0.33	0.26	0.002	0.36

**Table 3:** Signed mean and standard deviation mean surface difference (sMSD) and Hausdorff distance (sHD) comparing synovial tissue segmentations using 2D U-Net to 2D U-Net++. Also, the performance metric comparing the 3D reconstructions of the synovial volume based on the 2D U-Net and 2D U-Net++. P-values correspond to a comparison between 2D and 3D segmentation performance.

Segmentation	Method	sMSD(mm)	sHD(mm)
*2D Images*	U-Net	0.14 ± 0.22	0.75 ± 1.41
	U-Net++	0.15 ± 0.19	0.70 ± 1.09
	P-Value	0.92	0.89
*3D Reconstructed Images*	U-Net	−0.08 ± 0.04	0.32 ± 0.15
	U-Net++	−0.07 ± 0.06	0.31 ± 0.15
	P-Value	0.75	0.50

Computation time using the U-Net method was 0.029 s for each 2D segmentation, with an average of ten 2D US slices per 3D US volume. The 3D reconstruction time was 0.27 s resulting in a total mean 3D segmentation time of 0.56 s. For U-Net++, the computation time was 0.088 s for each 2D segmentation, and the average 3D reconstruction time was 0.27 s. This resulted in a total mean 3D segmentation time of 0.56 s. These processing times are significantly shorter than the time required to complete a manual 3D segmentation which was approximately 10 min.

**Fig. 5:**
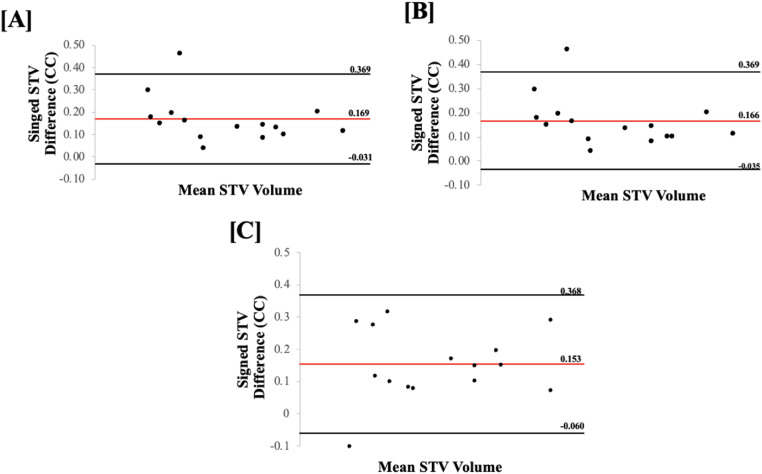
(A) Bland-Altman plot showing the fixed bias of the signed STV volume difference of the manual STV volume segmentations compared to the U-Net reconstructed STV volumes. (B) Bland -Altman plots indicating the fixed bias of the signed STV volume difference of the manual STV volume segmentations compared to the U-Net++ reconstructed STV volumes. (C) Bland-Altman plot showing the fixed bias of the signed STV volume difference of the manual STV volume segmentations of rater 1 compared to rater 2.

Table 4 shows results in 3D reconstruction segmentations compared to manual segmentations for the two different raters. The mean STV segmented by rater 1 was found to be 0.26 ± 0.13 cm [3], compared to 0.30 ± 0.14 cm [3] resulting from the algorithmic 3D reconstructions of the U-Net segmentations. This resulted in a mean VPD of 16.02 ± 10.2% between the manual 3D segmentations and the algorithmic reconstructions. The mean manual STV segmented by rater 2 was 0.32 ± 0.16 cm [3], while the mean volume for the algorithmic 3D reconstructions was 0.31 ± 0.14 cm [3]. The mean VPD between the manual segmentations and the reconstructed segmentations for rater 2 was 7.79 ± 7.58% (Fig. 5). A Wilcoxon matched pairs signed rank test indicated that the differences were statistically significantly different.

**Table 4:** Mean± standard deviation comparing manual segmentation from rater 1 and 2 to algorithmic 3D CMC1 synovial tissue segmentations.

Method	Rater	Precision (%)	Recall (%)	Accuracy (%)	DSC (%)	MSD (mm)	HD (mm)	A/VPD (%)
*U-Net*	Rater 1	81.1 ± 6.8	93.7 ± 3.6	96.16± 1.53	86.9 ± 4.8	0.14± 0.04	0.5 ± 0.1	16.9 ± 10.2
	Rater 2	79.5 ± 6.9	79.3 ± 6.8	93.71± 2.91	79.2 ± 5.5	0.22± 0.04	1.2 ± 0.6	7.8 ± 7.6
	P-Value	0.43	< 0.001	< 0.001	0.004	0.015	0.999	0.002

## Discussion

Segmentations of synovial tissue volume in CMC1 OA patients using 3D US provide a potential advancement in methods for imaging and monitoring disease progression, leading to opportunities for planning and monitoring treatment. 3D US provides an accurate and cost-effective method for assessing synovial tissue volumes. Unfortunately, this imaging method is limited by a time-consuming and often difficult manual segmentation process. To streamline workflow and reduce segmentation processing time, we have applied a previously developed 2D deep learning algorithm with a 3D reconstruction of the generated synovial tissue boundaries that allows accurate and precise estimation of the STV in the CMC1 joint.

The U-Net and U-Net++ algorithms both performed similarly for the 2D segmentation and the 3D reconstructions, demonstrating no preferred network when segmenting CMC1 OA images. A comparison of the mean segmentation performance indicated higher overall performance for the 3D reconstruction for both U-Net and U-Net ++. Hausdorf distance was the only metric that showed a statistically significant difference between U-Net and U-Net++ for the 3D reconstruction segmentations.

Previous work completed in our lab investigated the use of a U-Net algorithm for automatic segmentation of knee femoral cartilage and reported excellent agreement between manual 3D surface and algorithmic reconstructions, as well as highlighting limitations of the 3D reconstruction approach [21]. When using CNNs to segment 3D US images of knee femoral cartilage, Du Toit and Orlando reported that the reconstruction method produced rectangular surfaces, therefore cutting off regions of cartilage that could be observed in manual segmentations [21]. This resulted in lower performance metrics on 3D reconstruction segmentation compared to the 2D slice segmentations and manual segmentations observed in the study. When we applied a similar automated segmentation method to our CMC1 OA images, the previously reported limitation with cartilage size was not encountered due to the small size of the joint. In contrast to the knee images, we had to implement a user-defined region-of-interest to provide the algorithm with an enhanced focus on the target area, including the trapezium and the first metacarpal head. This method greatly reduced the size of the non-target areas, reducing the likelihood of mis-segmentation due to the narrowed focus of the algorithm. This is an important addition to our method as many structures commonly observed in 3D US images may have similar hypoechoic features associated with synovitis.

Synovial membrane hypertrophy is one of the pathophysiological changes associated with the progression of osteoarthritis, as mentioned previously. As membrane hypertrophy increases, it becomes increasingly difficult to identify the membrane boundaries due to similar coloration of the surrounding soft tissue. Our trained U-Net is robust to some amount of shadowing, as shown in the middle row where the algorithm accurately segmented the area of interest even in the presence of shadowing artifacts. In the bottom row, the lowest-performing case, the extensive synovial hypertrophy resulted in a smaller algorithmic segmentation that does not agree as closely with the manual gold standard. It is important to note that even in this poor-quality image, the algorithm does not confuse nearby anatomy with the synovial region of interest. This may be attributed to the selection of the region-of-interest.

The manual segmentations used for the training dataset were provided by rater 1, while the network did not receive any manual segmentations from rater 2 for training. Manual segmentations were moderately similar between raters with a percent difference of 19.94 ± 16.23%. This translates to an absolute synovial tissue volume of 1.17± 0.02 mm[3].

Automated segmentation performance was consistently lower for rater 2, with a Wilcoxon paired *t*-test showing statistically significant differences for all metrics except Hausdorff distance and precision. This highlights a limitation in the robustness of our algorithm to the manual rater. Manual segmentation of the synovial tissue volume of the CMC1 joint required on average 10–15 min. Our deep learning model required a total segmentation time of only 0.56 s, providing for a more rapid segmentation generation. It will require more training with a larger data set and segmentations provided by different raters to increase the robustness and generalizability of the algorithm.

While we have demonstrated promising performance of our automatic synovial tissue volume segmentation method, we must address several limitations associated with our training dataset. Although our testing dataset included manual segmentations from two different raters, the training dataset used the manual segmentations from only one rater. Significant differences were observed between all performance metrics between rater 1 and rater 2, except for precision (*p* = 0.43) and Hausdorff distance (*p* = 0.99). Future studies will focus on determining algorithm robustness with more raters and repeated measures testing.

The training set of 104 3D US images, which were resliced into 1040 2D US images, is a small dataset in the context of deep learning applications. Additionally, there was no diversity of US probes and US systems used or of image and voxel size, potentially limiting the generalizability of our current approach. To overcome this limitation, future work will focus on the addition of a larger, more diverse dataset, including data from multiple US machines, medical centers, raters, and patients at varying stages of osteoarthritis. Expansion of the current dataset would enable investigation into how OA pathophysiology influences algorithm performance metrics longitudinally as well as improve algorithm robustness and ultimately increase the potential for clinical use.

The greatest advantage of integrating deep learning technology into our 3D US system's image processing workflow is the potential to reconstruct patient STV rapidly, accurately, and precisely. The addition of our proposed algorithm would allow the volume reconstruction process to be conducted in a single imaging session without additional post-image processing or extensive additional training. The application of this network to the current 3D US imaging system decreases the segmentation processing time substantially from approximately 10–15 min for manual segmentation to 0.56 s per CMC1 joint. This provides users with a time-efficient method for evaluating synovial tissue volumes that is also safe, cost-effective and non-invasive. The clinical significance of the application of this deep learning method to our system is the impact it may have on the workflow of clinical trials. It would allow users to obtain and analyze 3D US images without having to manually segment image volumes and surfaces or significantly extend the examination time. Its integration would relieve many of the limitations associated with current methods for assessing synovial tissue health such as the qualitative grading scales used in 2D US.

## Declaration of competing interest

The authors have no relevant conflicts of interest to disclose.
